# The cultivable endophytic fungal community of *Scutellaria baicalensis*: diversity and relevance to flavonoid production by the host

**DOI:** 10.1080/15592324.2022.2068834

**Published:** 2022-05-08

**Authors:** Xiao-Xuan Cui, Lei Wang, Hui-Yong Fang, Yu-Guang Zheng, Chun-Yan Su

**Affiliations:** a International Joint Research Center on Resource Utilization and Quality Evaluation of Traditional Chinese Medicine of Hebei Province, Hebei University of Chinese Medicine, Shijiazhuang, Hebei, China; b Traditional Chinese Medicine Processing Technology Innovation Center of Hebei Province, Hebei University of Chinese Medicine, Shijiazhuang, Hebei, China; c Hebei Chemical and Pharmaceutical College, Shijiazhuang, Hebei, China

**Keywords:** *Scutellaria baicalensis*, endophytic fungi, community composition, diversity analysis, *Alternaria*, flavonoids

## Abstract

*Scutellaria baicalensis* (*SB*), a traditional Chinese medicinal plant, is widely used because of its important pharmacological activities. However, the endophytic fungi that promote flavonoid accumulation in *SB* remain unclear. Therefore, we analyzed the endophytic fungal community of *SB* and screened the endophytic fungi that might promote flavonoid synthesis in *SB*. ITS1/ITS4Blast was used to identify the endophytic fungi in *SB*. In total, 687 strains were identified in 57 genera. The dominant genus in the leaves and stems was *Alternaria* and that in the roots was *Fusarium. Alternaria* was the dominant genus in *SB* collected from all sites and in wild and cultivated *SB*. Alpha diversity indexes indicated more abundant endophytic fungi in samples from Chengde, the genuine producing area of *SB*, than in those from other sites. Beta diversity index analysis indicated that *SB* plants with closer geographical relationships showed more similar endophytic fungal community profiles. Spearman correlation analysis revealed that baicalin, wogonoside, wogonin, and oroxylin A contents were significantly correlated with the relative abundance of *Alternaria*. Overall, the results indicate the importance of geographical factors in influencing the endophytic fungal community of *SB* and suggest that the presence of *Alternaria* spp. might contribute to flavonoid synthesis in *SB.*

## Introduction

*Scutellaria baicalensis* (*SB*), a perennial plant of the Lamiaceae family, is widely grown in the northern part of China, whereas Chengde in Hebei Province is its genuine producing area. The root of *SB*, named as “*huangqin*” in China, is one of the most important traditional Chinese medicines. Clinically, *SB* roots are used to treat pneumonia, hypertension, jaundice, dysentery, intestinal catarrh, and pyogenic infection.^1^ In particular, baicalein and wogonin, the main bioactive compounds in *SB*, were also found to inhibit SARS-CoV-2 replication and to block the virus-binding sites.^2–5^ The leaves and stems of *SB* can improve memory impairments, neuronal injuries,^6^ and cognitive function, and delay the progression of dementia.^7^ Flavonoids and their derivatives, including baicalin, baicalein, wogonoside, wogonin, and oroxylin A, are the main bioactive components of *SB*.^1^

Endophytic fungi are considered as microorganisms that live inside cells or in the intercellular spaces of a plant during a certain life stage or throughout their life cycle, without showing any symptoms of disease.^8^ They are widely distributed among medicinal plants. The endophytic fungal community of a plant is influenced by its tissues, geographical locations, and growth conditions (wild or cultivated).^9–11^ Studies on the diversity and community composition of endophytic fungi in a given plant species may indicate that they provide ecological benefits to plants. This is particularly true when the host plants grow in different habitats. Moreover, endophytic fungi also promote the production of secondary metabolites in host plants by stimulating the key genes in their biosynthesis pathways,^12,13^ or by synthesizing enzymes that can transform precursors to active compounds or their analogs.^14^ Therefore, screening phytobeneficial endophytic fungi has become an important means of promoting the synthesis of bioactive metabolites.

In recent decades, owing to the scarcity of wild plant resources, cultivated *SB* has been used in clinical practice. Therefore, studying the endophytic fungal community of *SB* and screening endophytic fungi that can promote the accumulation of flavonoids in *SB* may positively influence improvement in the quality of cultivated *SB*. However, the endophytic fungi that promote flavonoid accumulation in *SB* remain unclear. Therefore, this study focused on the endophytic fungal community composition of wild and cultivated *SB* and the correlation between the endophytic fungal community and flavonoid content to screen endophytic fungi that might promote flavonoid synthesis in *SB*.

## Materials and methods

### Sample collection

Healthy wild and cultivated *SB* samples were collected from five sampling sites in Hebei Province, China (Table 1). All samples were stored in ice boxes and immediately sent to the laboratory for further analysis. The samples were then separated into two parts. One part was used to determine the flavonoid content, and the other part was used for endophytic fungal isolation.

**Table 1.** Sample information of *Scutellaria baicalensis.*

Code No.	Location	Growth Type	Growth time	Sampling site,altitude	Number
CLY	Chongli county,	Wild	Unknown	115°25′44.39″E, 41°02′30.76″N,1382 m	25
	Zhangjiakou city				
CDY	Chengde county,	Wild	Unknown	118°14′50.32″E, 41°01′39.50″N, 60 m	18
	Chengde city				
LHY	Longhua county,	Wild	Unknown	117°27′29.16″E, 41°20′09.24″N, 971 m	24
	Chengde city				
LHZ	Longhua county,	Artificial cultivation	3 years	117°27′38.88″E, 41°19′48″N, 943 m	12
	Chengde city				
WDZ	Wangdu county,	Artificial cultivation	2 years	115°08′52.79″E, 38°44′57.22″N, 44 m	31

### Isolation of endophytic fungi

Fresh *SB* samples were rinsed thoroughly with running water for 10 min; the roots, stems, and leaves were then cut into 5 mm × 5 mm pieces. For each section, six tissue segments were randomly selected for removal of surface microbes. All the tissues underwent the following surface sterilization steps: soaking in sterile water for 1 min followed by 75% ethanol for 1 min. The leaves were then soaked in 3.5% sodium hypochlorite for 1 min, and the roots and stems were soaked for 3 min. Further, all tissues were soaked in 75% ethanol for 1 min and rinsed with sterile water for 1 min. Finally, the surface water was removed using sterilized filter paper, and fresh cuts were made using a sterilized scalpel. Sets of three segments were then evenly placed on a 90 mm Petri dish containing potato dextrose agar (PDA) medium. The plates were incubated at a constant temperature of 26°C for 5–7 days. The fungal colonies were transferred to fresh PDA media for purification. Purified fungi were kept in 15% glycerin and store at −80°C for further identification.

### Identification of endophytic fungi

The Fungi Genomic DNA Extraction Kit (Solarbio Science & Technology Co., Ltd, Beijing, China) was used to extract genomic DNA from endophytic fungi, according to the manufacturer’s protocol. From the extracted DNA, 18S rDNA was amplified by polymerase chain reaction (PCR) using the primers ITS1 (5′-TCCGTAGGTGAACCTGCGG-3′) and ITS4 (5′-TCCTCCGCTTATTGATATGC-3′). PCR was performed in a 30 μL mixture containing 15 μL of 2× Taq PCR Mix (Generay Biotech Co., Ltd., Shanghai., China.), 1.0 μL of each primer (1.0 μM), 1 μL of template DNA, and 12 μL of ddH_2_O.

The thermal cycling conditions were as follows: denaturation at 94°C for 5 min, followed by 37 cycles of denaturation at 94°C for 30s, annealing at 55°C for 40s, and extension at 72°C for 30s. Finally the reaction mixture was maintained at 72°C for 7 min. The resulting PCR products were sequenced by Sangon Biotech (Shanghai, China). Each sequence was used as a query sequence to search for similar sequences using BLAST (https://blast.ncbi.nlm.nih.gov/Blast.cgi) in GenBank.

### Extraction and quantification of flavonoids from SB

*SB* samples were dried at 50°C to a constant weight. The dried samples (leaves, stems, and roots) were comminuted and passed through 60-mesh sieves. Accurately weighed (18.75 mg) sample powders were extracted using 1.5 mL of 60% ethanol by ultrasonication for 40 min and then centrifuged at 13000 rpm for 10 min. The supernatant was then collected. The sample solution was filtered through a 0.22 μm pore-size polytetrafluoroethylene filter.

The extracts were analyzed on a Diamonsil C18 (4.6 × 250 mm, 5.0 µm, DiKMA, Beijing, China) column connected to a SHIMADZU LC-20AD high-performance liquid chromatography (HPLC) system (SHIMADZU, Kyoto, Japan). The mobile phases comprised A (water containing 0.1% formic acid) and B (acetonitrile). The gradient elution program was as follows: 25% B at 0–10 min, 25–45% B at 10–30 min, 45–55% B at 30–55 min. The detection wavelength was set at 254 nm. The sample injection volume was 10 μL and the column temperature was maintained at 25°C.

### Data analysis

Alpha diversity, including the Shannon index, Simpson index, abundance-based coverage estimator (ACE), and Chao1 index; and beta diversity, including principal coordinate analysis (PCoA) and the unweighted pair-group method with arithmetic means (UPGMA), were determined using QIIME and displayed using R software. The contents of flavonoids were expressed as mean±SD from three separate observations. The data analyzed by one-way analysis of variance (ANOVA) Duncan’s multiple range tests using the SPSS19.0 software. Endophytic fungi with relative abundance higher than 5% and which were found in common for at least 3 sites, were chosen for further correlation analysis. Spearman correlation analysis between the relative abundance of endophytic fungi and flavonoid content in *SB* was performed using Origin 2021.

## Results

### Fungal community composition

#### Fungal community composition in different organs of SB

**Figure 1.**
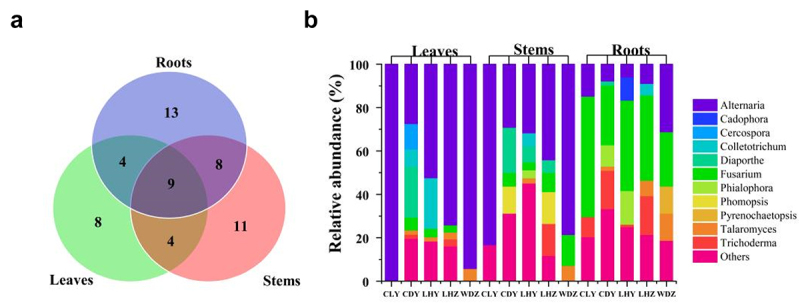
The endophytic fungal community composition in organs of *SB* at the genus level. (a) Venn diagram of different organs of *SB*. The numbers inside the diagram indicate the number of genera. (b) The relative abundance of endophytic fungi in different organs of *SB*; endophytic fungi with relative abundances lower than 5% are indicated as “Others”.

In total, 687 endophytic fungal strains were isolated from 1980 tissue segments. The highest number of strains was found in the roots (261), followed by the leaves (230) and stems (196). According to ITS sequence analysis, the fungal strains belonged to 57 genera. As depicted in Figure 1a, 32 genera were found in the stems, followed by the roots (31) and leaves (23). Nine genera were common to all three organs, whereas 13, 11, and 8 genera were specific to the roots, stems and leaves, respectively (Additional Table S1).

As demonstrated in Figure 1b, *Alternaria* was dominant in all leaves, with a relative abundance ranging from 27.4% (CDY) to 100% (CLY). Similarly, *Alternaria* was dominant in all stems, with a relative abundance ranging from 29.1% (CDY) to 83.3% (CLY). However, in the roots, *Fusarium* was dominant in CLY, LHY, LHZ, and CDY, with the relative abundances ranging from 27.4% (CDY) to 55.5% (CLY), whereas *Alternaria* was dominant in WDZ, with a relative abundance of 31.25%.

#### Fungal community composition in SB from different sampling sites

**Figure 2.**
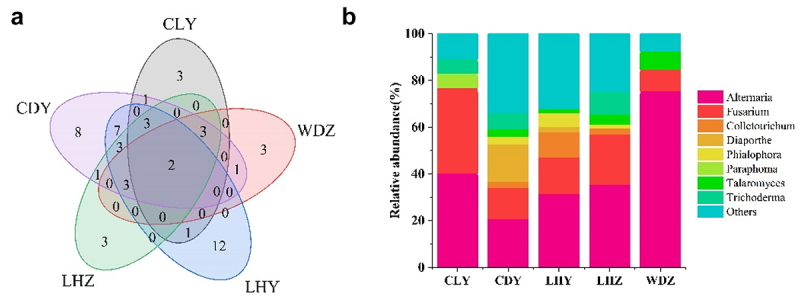
The endophytic fungal community composition of *SB* from different sampling sites, at the genus level. (a) Venn diagram of the endophytic fungi of *SB* from different sampling sites. The numbers in the diagram indicate the number of genera. (b) The relative abundance of endophytic fungi of *SB* from different sites; the endophytic fungi with relative abundances lower than 5% are indicated as “Others”.

The highest number of strains was obtained from LHY (269), followed by CDY (150), LHZ (121), CLY (82), and WDZ (65). According to ITS sequence analysis, 37 genera were found in LHY, followed by CDY (35 genera), LHZ (21 genera), CLY (11 genera), and WDZ (7 genera). As depicted in Figure 2a, two genera (*Alternaria, Fusarium*) were commonly found at all five sampling sites; further, 3, 3, 12, 3, and 8 genera were specific to CLY, WDZ, LHY, LHZ, and CDY, respectively (Additional Table S2).

As shown in Figure 2b, *Alternaria* was dominant at all sampling sites, with a relative abundance ranging from 20.6% (CDY) to 75.3% (WDZ).

#### Fungal community composition of wild and cultivated SB

**Figure 3.**
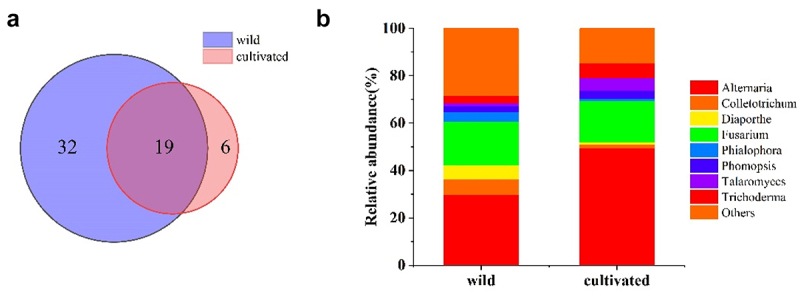
Endophytic fungal community composition of wild and cultivated *SB*, at the genus level. (a) Venn diagram of endophytic fungi in wild and cultivated *SB*. The numbers inside the diagram indicate the number of genera. (b) The relative abundance of endophytic fungi in wild and cultivated *SB*; endophytic fungi with relative abundances lower than 5% are indicated as “Others”.

In total, 501 endophytic fungal strains were obtained from wild *SB*, whereas 186 strains were obtained from cultivated *SB*. As depicted in Figure 3a, according to ITS sequence analysis, endophytic fungi obtained from wild *SB* were assigned to 51 genera, and endophytic fungi obtained from cultivated *SB* were assigned to 25 genera. 19 genera were common between wild and cultivated *SB*, whereas 32 and 6 genera were specific to wild *SB* and cultivated *SB*, respectively (Additional Table S3).

As shown in Figure 3b, the dominant genera in wild and cultivated *SB* were all *Alternaria*, with relative abundances of 29.7% and 49.4%, respectively.

### Diversity analysis

#### Alpha diversity analysis

The alpha diversity indices of *SB* from five sampling sites are listed in Table 2. The highest Shannon, Chao1, ACE, and the lowest Simpson indices were found in CDY, indicating a higher diversity of endophytic fungi in CDY. In contrast, the lowest Shannon, Chao1, and ACE, and the highest Simpson indices were found in WDZ, indicating a low diversity of endophytic fungi in WDZ.

**Table 2.** Alpha diversity indices of *SB* samples

Sample Name	Shannon	Simpson	Chao1	ACE
CLY	1.51	0.29	26	29.85
CDY	2.81	0.093	57.66	75.92
LHY	2.56	0.14	48.66	67.01
LHZ	2.18	0.18	24	26.53
WDZ	0.93	0.57	8.5	10.6

#### Beta diversity analysis

**Figure 4.**
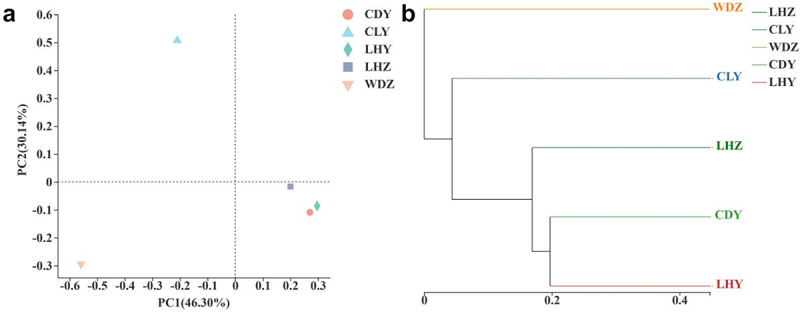
Principal coordinate analysis (PcoA) analysis and the Unweighted pair Group Method with Arithmetic mean (UPGMA) of the Binary jaccard algorithm at the genus level. (a) PcoA analysis (b) UPGMA hierarchical cluster analysis.

Principal coordinate analysis (PCoA) based on Binary Jaccard was conducted to determine the relationship between different samples of *SB* (Figure 4a). The results showed that the first axis explained 46.30% of the data variability and the second axis explained 30.14%. The distance between CDY and LHY was the smallest, whereas the WDZ sample had the largest distance from the other samples. The unweighted pair-group method with arithmetic mean (UPGMA) was used to cluster the five samples based on the binary Jaccard (Figure 4b). The results revealed that CDY and LHY were clustered together, whereas WDZ had the largest difference compared with other samples in terms of fungal diversity, similar to the results of the PcoA.

#### The contents of five flavonoids in SB

**Figure 5.**
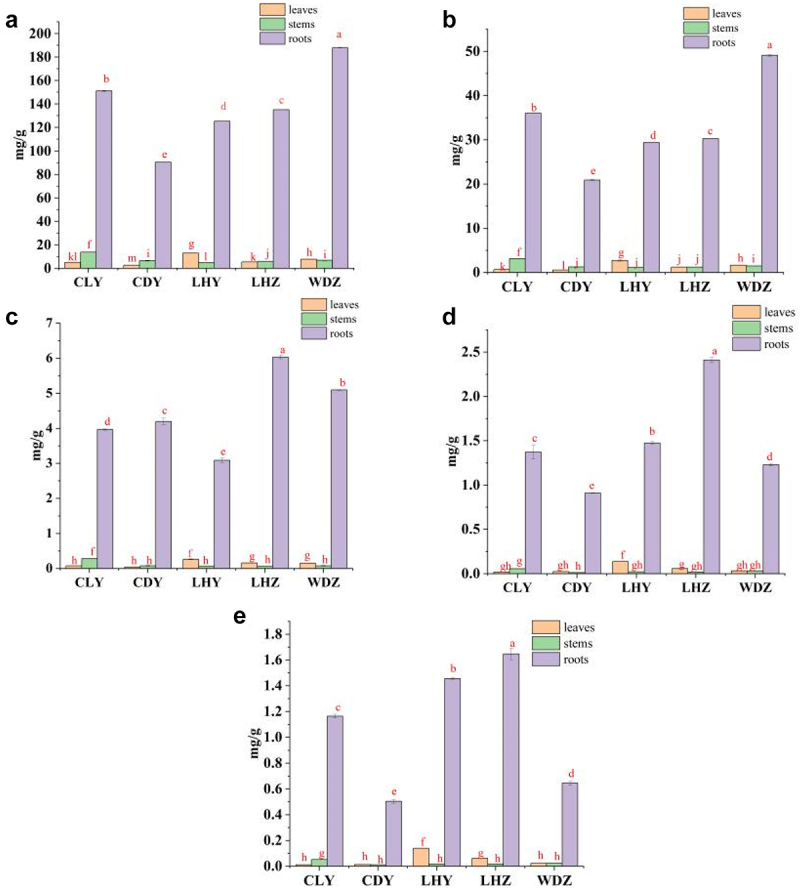
Contents of flavonoids in *SB*. (a–e) represent baicalin, baicalein, wogonoside, wogonin, and oroxylin A, respectively.

The flavonoid contents of *SB* are shown in Figure 5. The contents of baicalin, baicalein, wogonoside, wogonin, and oroxylin A in the roots were higher than those in the leaves and stems from all sampling sites. The contents of baicalin and wogonoside in the leaves from CLY and CDY were significantly higher than those in the stems (P < .05), whereas those in the stems from LHY and WDZ were significantly higher than those in the leaves. However, the baicalin and wogonoside contents in the leaves and stems of LHZ were slightly different. The contents of baicalein and oroxylin A in stems from CLY were significantly higher than those in leaves, whereas those in leaves from LHY and LHZ were significantly higher than those in stems. However, the contents of baicalein and oroxylin A in the leaves and stems from WDZ were not different. The content of wogonin in the stems of LHY was higher than that in the leaves; however, the content of wogonin in leaves and stems from other sampling sites was not different.

Based on our results, the highest content of total flavonoids in the roots of *SB* was found in WDZ (244.01 mg/g), followed by CLY (193.72 mg/g), LHZ (175.59 mg/g), LHY (160.75 mg/g), and CDY (116.94 mg/g).

Means within the same column with different letter marks among content level groups are significantly different

#### Correlation analysis between the endophytic fungi and metabolites in SB

**Figure 6.**
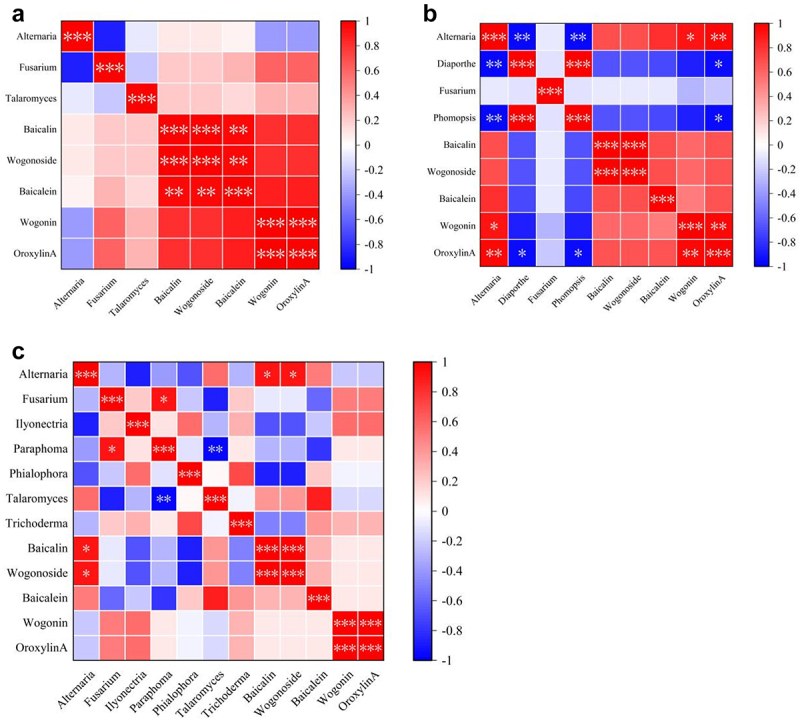
Heatmap of correlation between the relative abundance of endophytic fungi and flavonoids contents, (a–c) represent the contents in the leaves, stems, and roots, respectively. *: P < .05; **: P < .01; ***: P < .001.

To screen for potential endophytic fungi that can promote flavonoid biosynthesis in *SB*. Spearman analysis was used to determine the correlation between the relative abundance of endophytic fungi and the flavonoid content in *SB* (Figure 6). The results showed no significant correlation between the dominant endophytic fungal genus and flavonoids (A) in the leaves. Interestingly, in stems, the contents of wogonin (P < .05) and oroxylin A (P < .01) showed a significant positive correlation with the relative abundance of *Alternaria*, whereas the oroxylin A content showed a significantly negative correlation with the relative abundance of *Diaporthe* and *Phomopsis* (P < .01). Furthermore, in the roots, the contents of baicalin and wogonoside showed a significant positive correlation with the relative abundance of *Alternaria* (P < .05).

## Discussion

*SB* and its relevant preparations are widely used because of its medicinal value. In recent years, the natural resources of SB have been deficient and gradually replaced by cultivated plants, resulting in expansion of the planting area and increased production.^15^ Endophytic fungi have been suggested to promote the biosynthesis of secondary metabolites in host plants.^16–18^ Therefore, screening endophytic fungi that can promote host plant secondary metabolite biosynthesis is an effective way to improve the host quality. Our study thus focused on the endophytic fungal community composition of wild and cultivated *SB* collected from five sampling sites in Hebei Province, China. The correlation between the endophytic fungal community and flavonoids of host plants in different organs was analyzed to screen endophytic fungi that might promote host plant flavonoid synthesis.

In the current study, *Alternaria* was dominant in all the leaves and stems of *SB*, consistent with the results of previous studies.^19,20^ We found that *Fusarium* was dominant in the roots of all samples except WDZ, consistent with the findings of Li et al.^19^ and Ji et al.,^20^ who reported that *Fusarium* is one of the dominant genera in the roots of *SB*. Several *Fusarium* species are pathogens that cause root diseases in *SB*.^21^ Therefore, our results indicate the importance of prevention and control measures for root diseases caused by *Fusarium* during the growth of *SB*. Meanwhile, we found that *Alternaria* was dominant in the *SB* collected from all sampling sites, which is consistent with previous studies.^19,20^ Further, *Alternaria* was dominant in wild and cultivated *SB*, consistent with the findings of Li et al.^19^ and Ji et al.,^20^ who reported the dominance of *Alternaria* in wild and cultivated *SB*. Our results revealed that the endophytic fungi of *SB* showed organ, region, and growth pattern specificity, which has also been observed in other plants.^22–24^

Our results showed that CDY, LHY, and LHZ had higher alpha diversity indices, whereas the other two samples had relatively low alpha diversity indices. Notably, CDY, LHY, and LHZ were all collected from Chengde city, which is the genuine producing area of *SB*, while the other two samples were collected from non-genuine producing areas, indicating that the endophytic fungi of *SB* growing in genuine producing areas were more abundant; this phenomenon was also found in *Glycyrrhiza uralensis*,^25^ *Scrophularia ningpoensis* Hemsl,^26^ *Ligusticum chuanxiong* Hort.^27^ Further, the results of PcoA and UPGMA also suggested that endophytic fungi in *SB* collected from closer sampling sites had similar community compositions, whereas those in *SB* collected from distant sampling sites had different community compositions.^28,29^ Interestingly, we found that all endophytic fungi in the wild samples did not have a higher alpha diversity index than that of cultivated samples. Our results indicated that growth pattern had little effect on the diversity of endophytic fungi, consistent with the results of Chen et al.^30^

In our study, the five flavonoids (baicalin, wogonoside, baicalein, wogonin, and oroxylin A) in the roots of *SB* were significantly higher than those in the stems and leaves of all samples, consistent with the results of Shen et al.^31^ These five flavonoids have relatively high contents in *SB* roots.^32^ Our results also indicate that the contents of five flavonoids in the roots of wild *SB* were not higher than those in cultivated *SB*, consistent with the results of Guo et al.^33^ The flavonoid contents in the roots of *SB* grown in genuine producing areas were not higher than those in *SB* grown in non-genuine producing areas, consistent with the findings of Li et al.^34^ These results also suggest that it is unscientific to evaluate the quality of *SB* based only on flavonoid content.

Previous studies have already demonstrated that some endophytic fungi and their host plants have established a special relationship that can significantly influence the formation of secondary metabolic products in plants, thereby affecting the quantity of crude drugs derived from medicinal plants.^35^ This study investigated the relationship between the endophytic fungal community and baicalin, wogonoside, baicalein, wogonin, and oroxylin A in *SB*. Spearman’s correlation analysis showed that baicalin, wogonoside, wogonin, and oroxylin A in *SB* were positively correlated with the relative abundance of *Alternaria*. We thus speculated that *Alternaria* spp. might contribute to flavonoid biosynthesis in *SB*. Several *Alternaria* species are known to produce flavonoids in their host plants. Shi et al.^36^ found that *Alternaria* sp. MG1, isolated from *Vitis vinifera* L. cv. Merlo has a stable and high resveratrol-producing capability. Kou et al.^37^ isolated three *Alternaria* species that produces flavonoids in *Cyclocarya paliurus* (Batal.) Iljinskaja.

To our knowledge, this is the first report describing the diversity and community composition of culturable endophytic fungi in wild and cultivated *SB* collected from five sites in Hebei province, China. Our results indicated that *Alternaria* was the dominant genus in leaves and stems in all samples, whereas *Fusarium* was dominant in the roots of all samples except WDZ. *Alternaria* was the dominant genus in *SB* from all sampling sites, and *Alternaria* was the dominant genus in wild and cultivated *SB*. Alpha diversity and beta diversity analyses revealed that the producing area was one of the main factors affecting the diversity and community composition of endophytic fungi in *SB*. The contents of the five flavonoids in the roots of *SB* were higher than those in the leaves and stems. The flavonoid content in the roots was not affected by production area or growth pattern. Further, we speculated that *Alternaria* spp. might contribute to the accumulation of flavonoids in *SB*. Subsequently, based on these results mentioned above, we plan to use traditional co-culture or elicitor induction experiments in future studies to screen specific *Alternaria* strains that can promote flavonoid synthesis in the host.
